# Supplementary material for: Single-cell RNA-sequencing uncovers transcriptional states and fate decisions in haematopoiesis
Source: Nat Commun. 2017 Dec 11;8:2045. doi: 10.1038/s41467-017-02305-6 (PMC5725498; doi:10.1038/s41467-017-02305-6)
Supplement: Supplementary file 1 — Supplementary Information [file 41467_2017_2305_MOESM1_ESM.pdf]

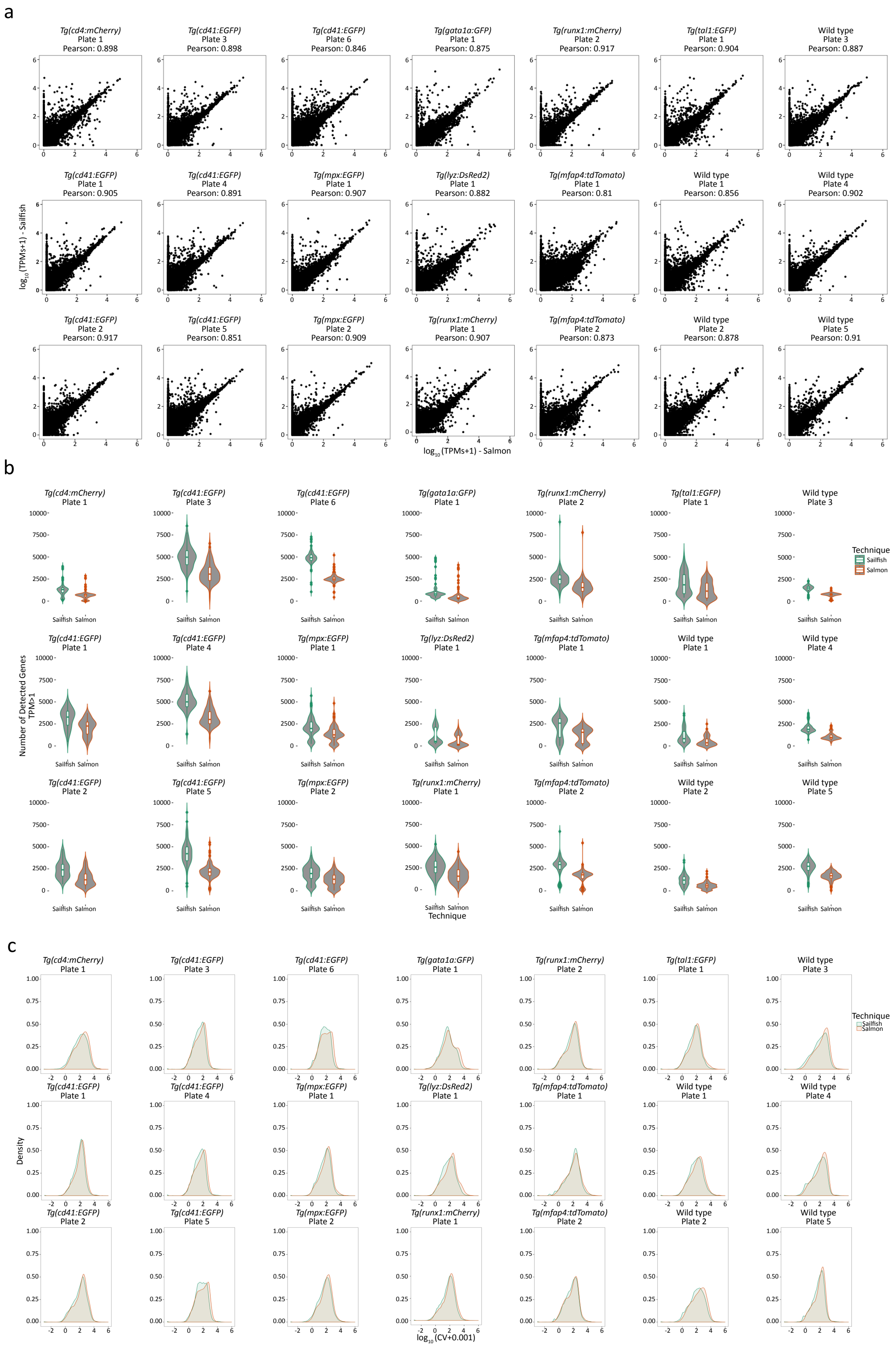

Supplementary Figure 1. Comparison of the difference between unique and multi-mapped alignment methods on our scRNA-Seq data set. a) Pearson correlation of the average gene expressions between Salmon and Sailfish. Gene quantification accuracy was assessed by selecting for each of the 21 sequenced plates the average $\log_{10}$ (TPM+1) gene expressions. b) Violin plots of the number of detected genes (TPM>1) at single-cell level. Salmon (unique mapped reads) and Sailfish (multi-mapped reads) were compared for each of the 21 plates. c) Distribution of the Coefficient of Variation. Comparison of log10(CV+0.001) gene expression (TPMs) values at a single-cell level between Salmon (unique mapped reads) and Sailfish (multi-mapped reads), across 21 plates.

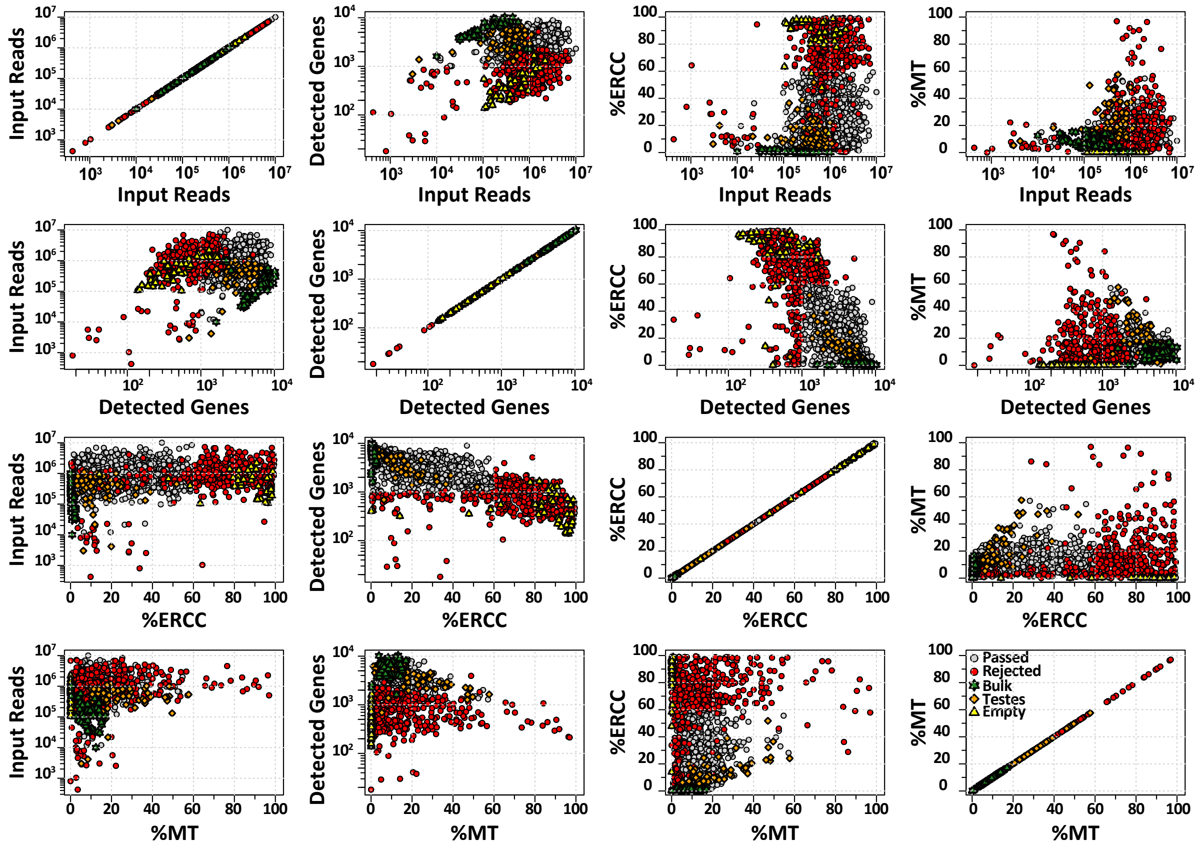

Supplementary Figure 2. Quality control of scRNA-seq data. Plots for the number of input reads, detected genes, %ERCCs and % mitochondrial (MT) genes on the y-axis in columns versus these four parameters on the x-axis in rows. The key in the bottom right hand plot indicates cells that have passed the quality control (% ERCCs <60%, mt <60%, at least 1000 detected genes), those that have failed and controls (bulk cells, empty wells and testes).

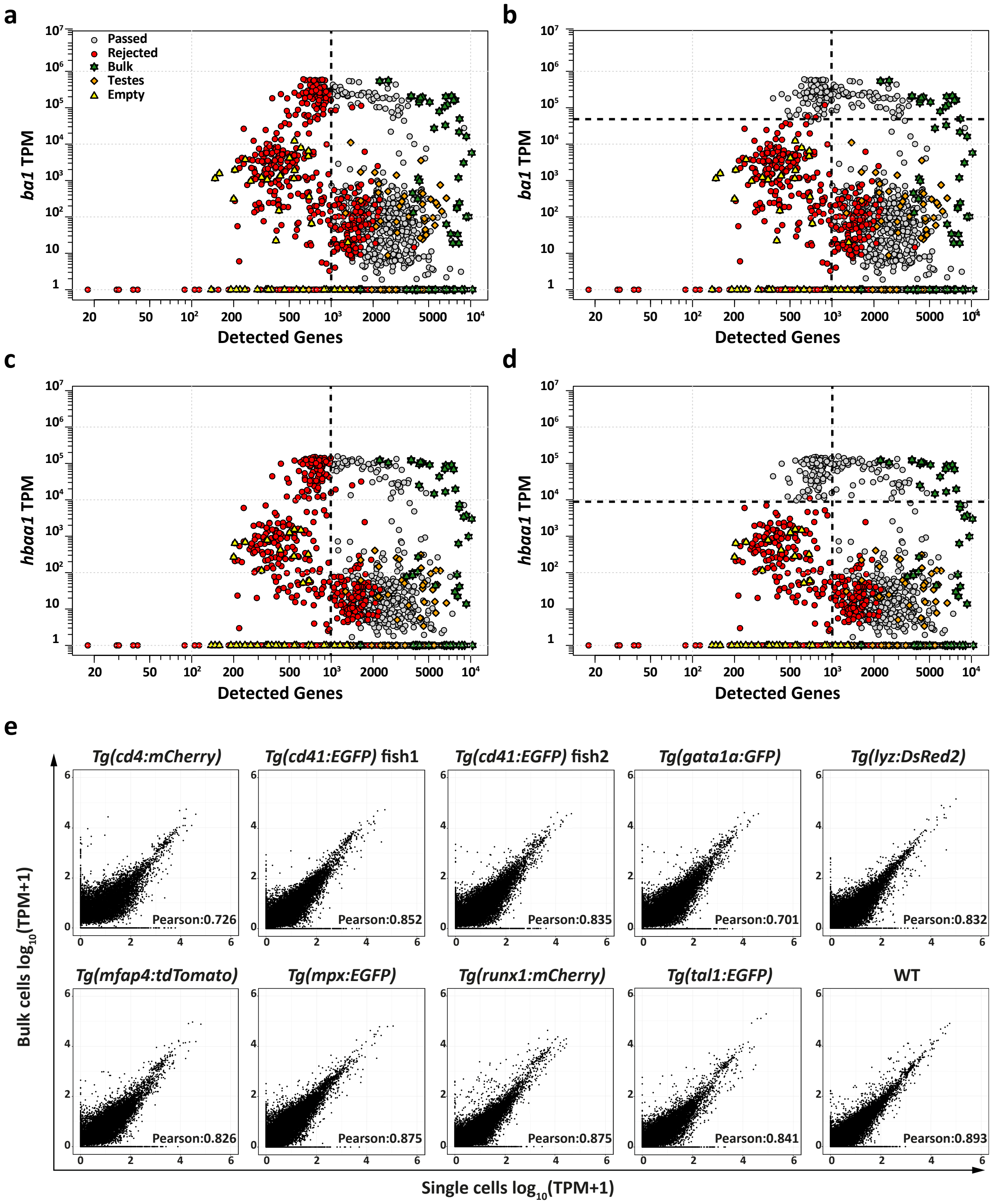

Supplementary Figure 3. Quality control of scRNA-seq data in the erythroid lineage and comparison of bulk versus single cell transcriptomes. The expression of the erythroid specific genes *ba1* and *hbaa1* were taken into account for quality control. a) and (c) plots show that many of the cells that initially failed QC (see key in a) have high expression of *ba1* (a) and *hbaa1* (c). These cells were therefore reassessed and those with >40000 *ba1* TPM (B) or >9000 *hbaa1* TPM (d) were included in the dataset. e) Correlation of average single cell transcriptome profiles and corresponding bulk wells for each fish line. The Pearson correlation coefficient shown in each plot indicates a strong correlation (0.7-0.9) between single and bulk transcriptome profiles.

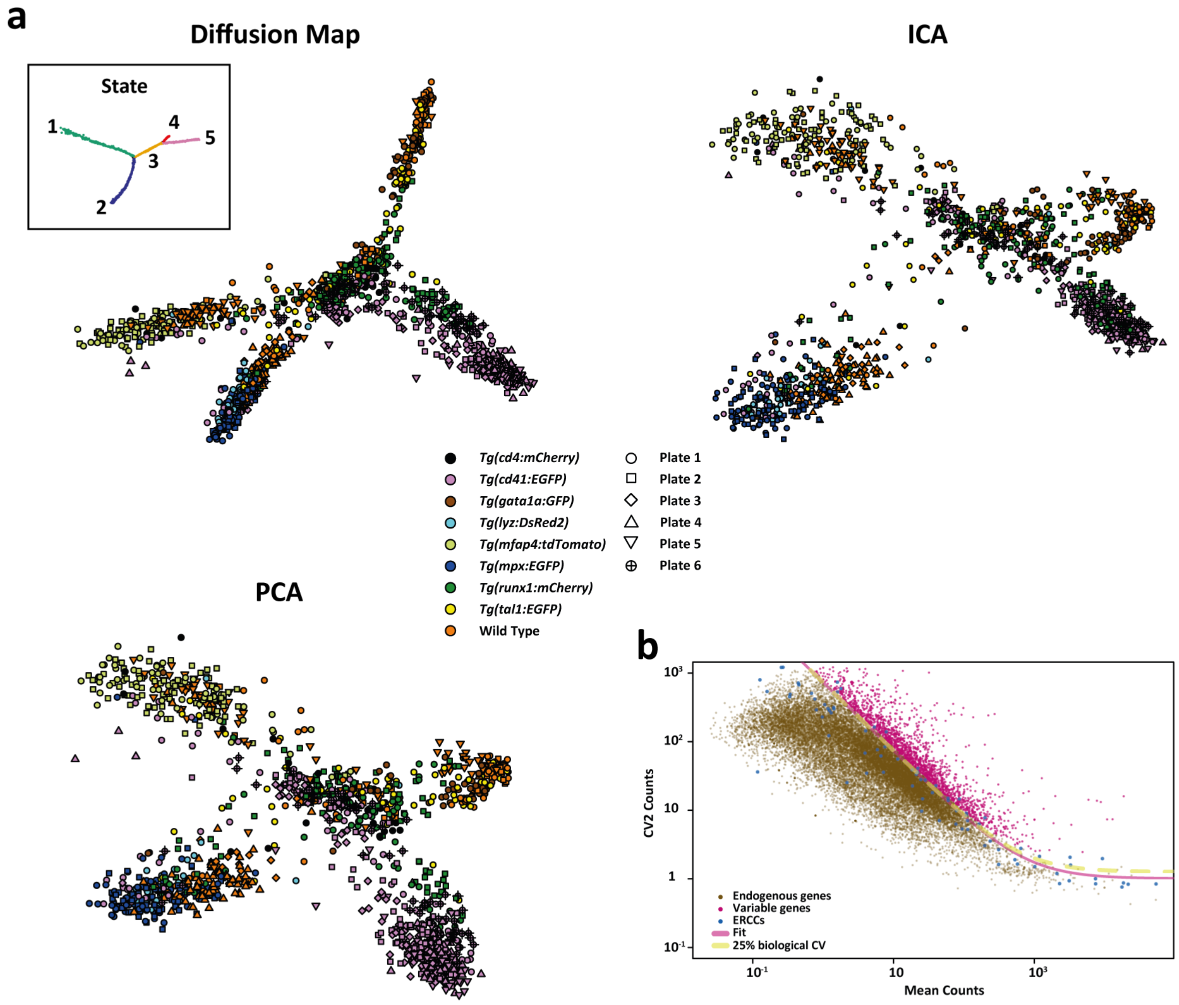

Supplementary Figure 4. Representation of single cell transcriptomes in three dimensional component space and the identification of the most highly variable genes. a) The 1,845 most highly variable genes were used to generate a diffusion map, independent component analyses and principal component analyses. The approximate positions of the cell states identified by Monocle 2 (Figure 1) are shown in the insert. Cells were derived from the transgenic lines and plates as listed in the key. b) The graph shows the squared coefficient of variance (CV^2) plotted versus mean read counts. The solid magenta line shows the curve of the technical noise fit and the dashed yellow line shows the position of genes with 25% biological CV. Blue dots indicate the ERCCs; magenta dots indicate the significantly variable genes; brown dots show the rest of genes expressed in the dataset.

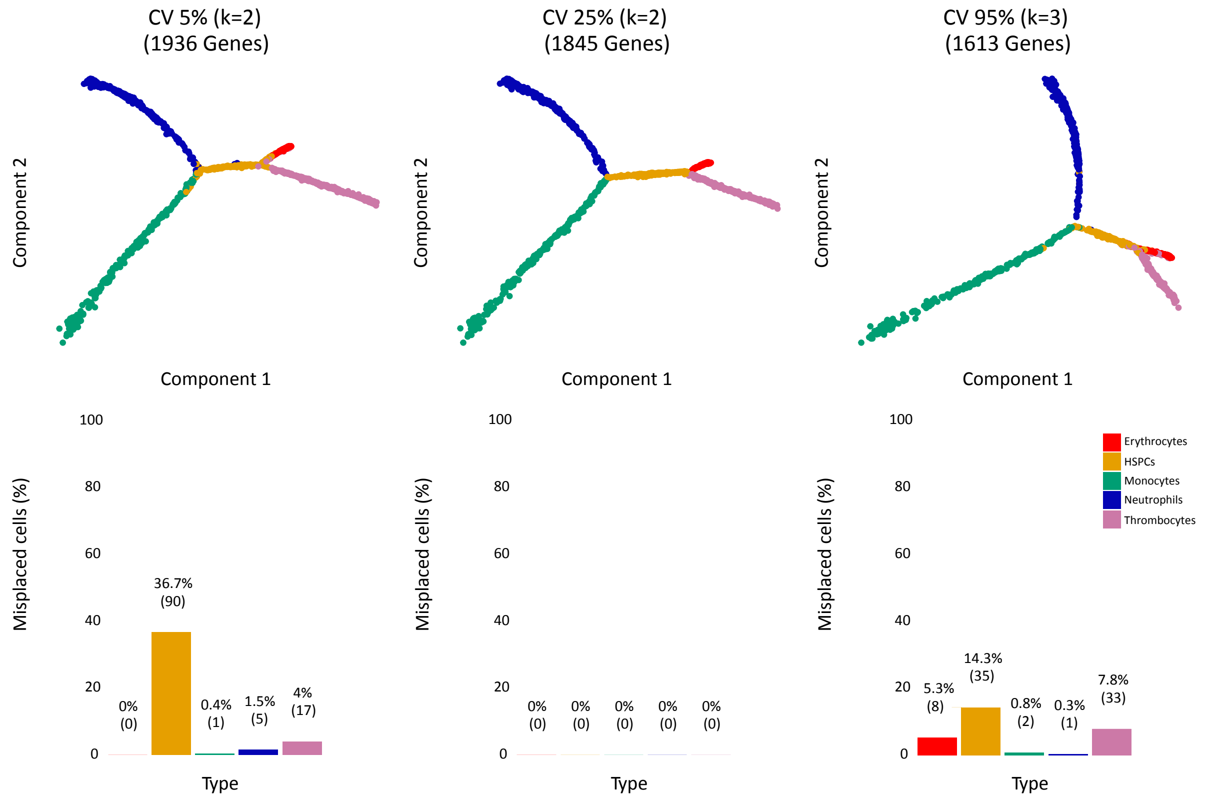

Supplementary Figure 5. Monocle trajectories generated using different sets of highly variable genes. Trajectories were generated using three different sets of highly variable genes. Highly variable genes were calculated using thresholds of 5%, 25%-(default) and 95% biological variation. For each reconstructed tree, the percentage (and a total number) of "misclassified" cells in each branch was calculated compared to the default setup (25%). k=number of components used.

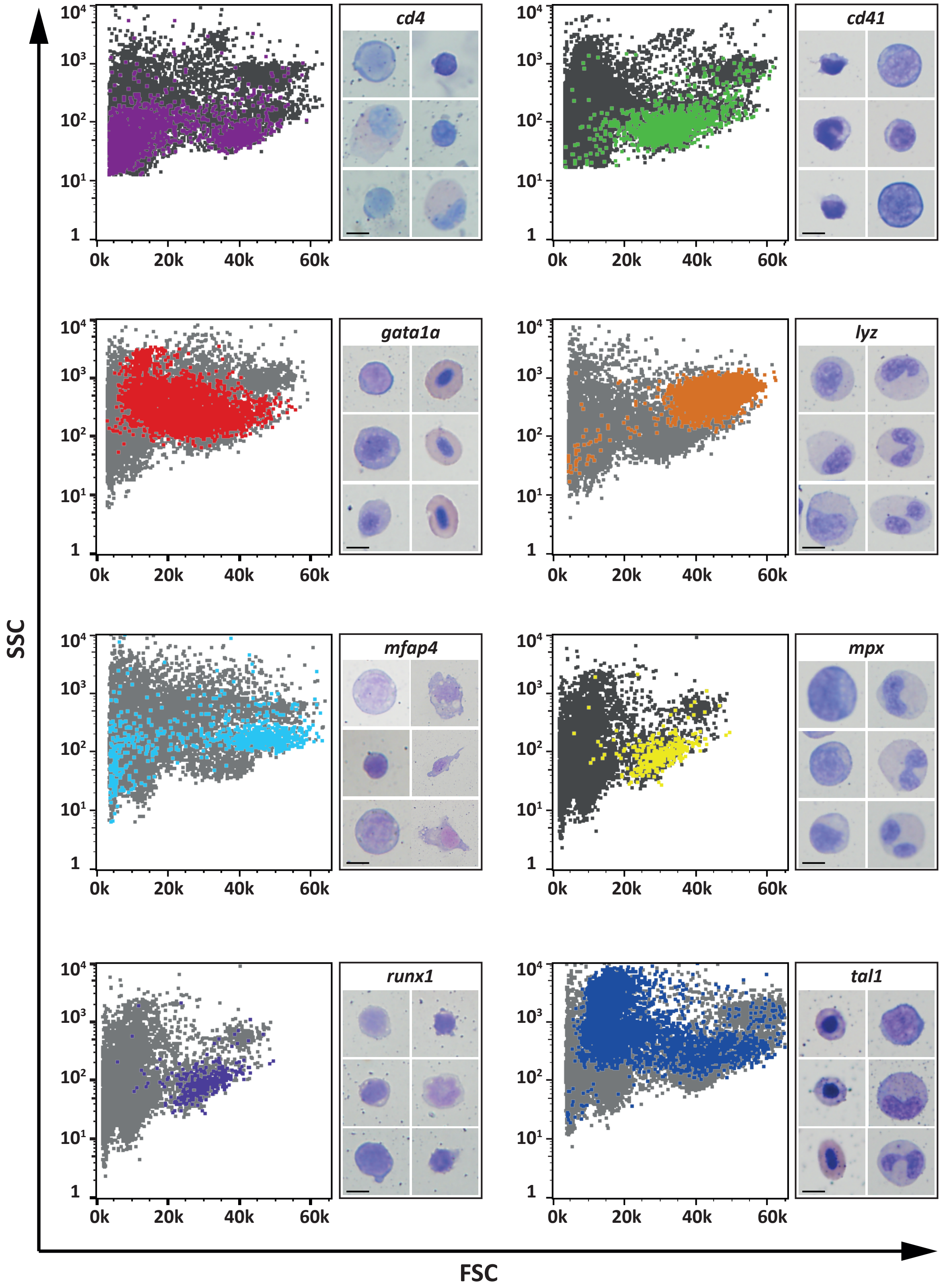

Supplementary Figure 6. The isolation and morphological characterisation of transgenic cell types. Representative FACS plots of cells isolated for scRNA-seq from each transgenic line. All cells that were positive for the fluorescent transgene were plotted on to forward/side scatter plots of live cells and are shown as coloured dots. To the right of each plot are the names of the genes and representative cells that were isolated by cytospins and stained with May-Grünwald Giemsa.

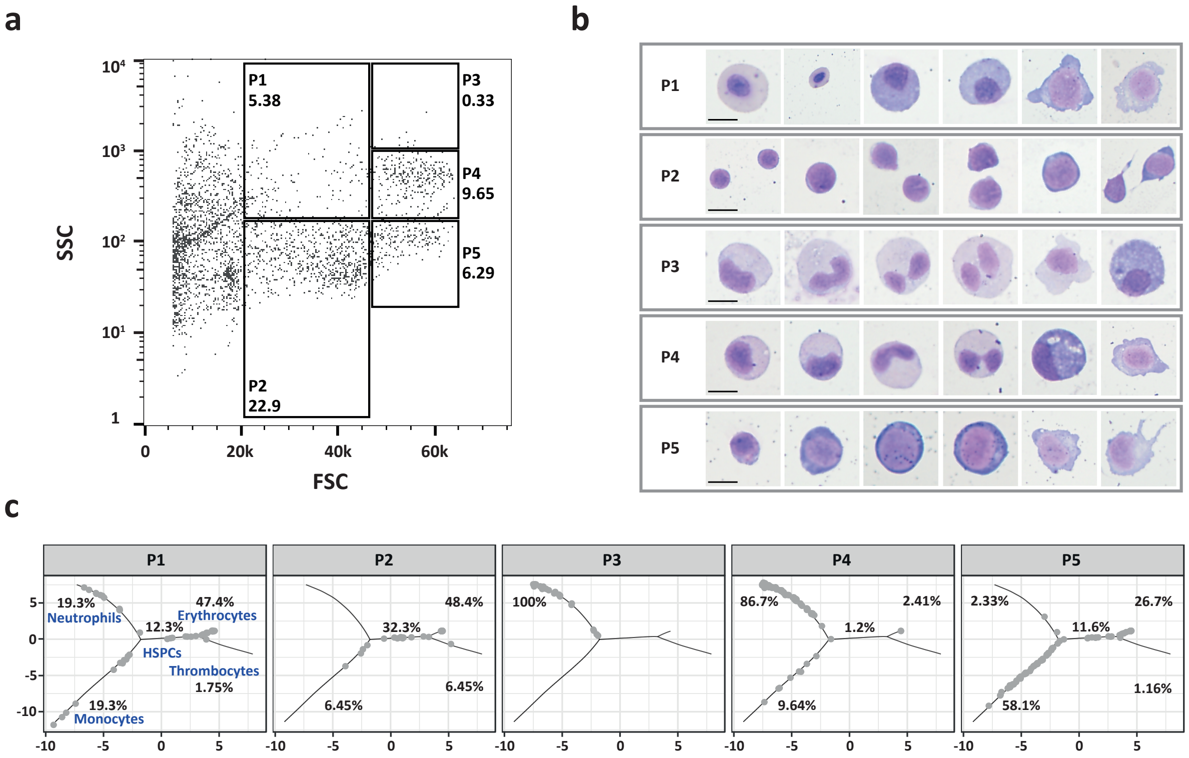

Supplementary Figure 7. The morphological characteristics of wild type cell sub-populations and their distribution in Monocle2. a**)** Flow cytometry forward scatter/side scatter plot of the wild type whole kidney marrow, showing the gating strategy for isolating populations P1-5. The percentage of live cells in each gate is also given. The cells on the left hand side of the P1 and P2 were gated out because the majority of these cells are erythrocytes. b) The cytospins of the representative cells from P1-P5 stained with May-Grünwald Giemsa. c) The trajectories of cell states predicted by Monocle are shown in grey for P1-P5, with the associated cell types labelled in blue. The percentage of cells from each sub-population contributing to each state is given next to the relevant trajectory.

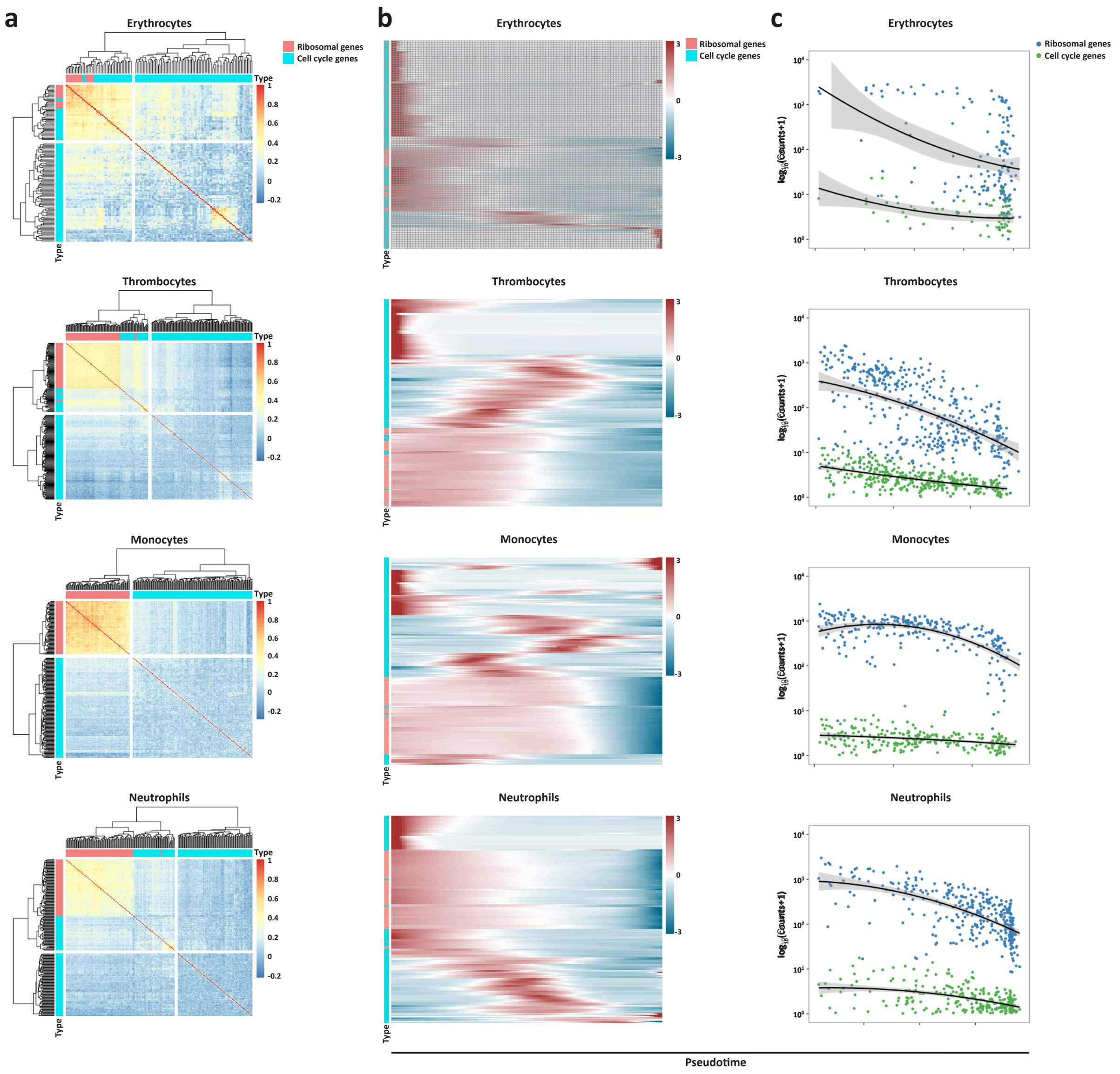

Supplementary Figure 8. Correlation analysis between ribosomal and cell cycle related genes. (a) Correlation heatmaps across all ribosomal and cell cycle genes, (b) correlation heatmaps of ribosomal and cell cycle genes in pseudotime and (c) average expression patterns of ribosomal and cell cycle genes in pseudotime.

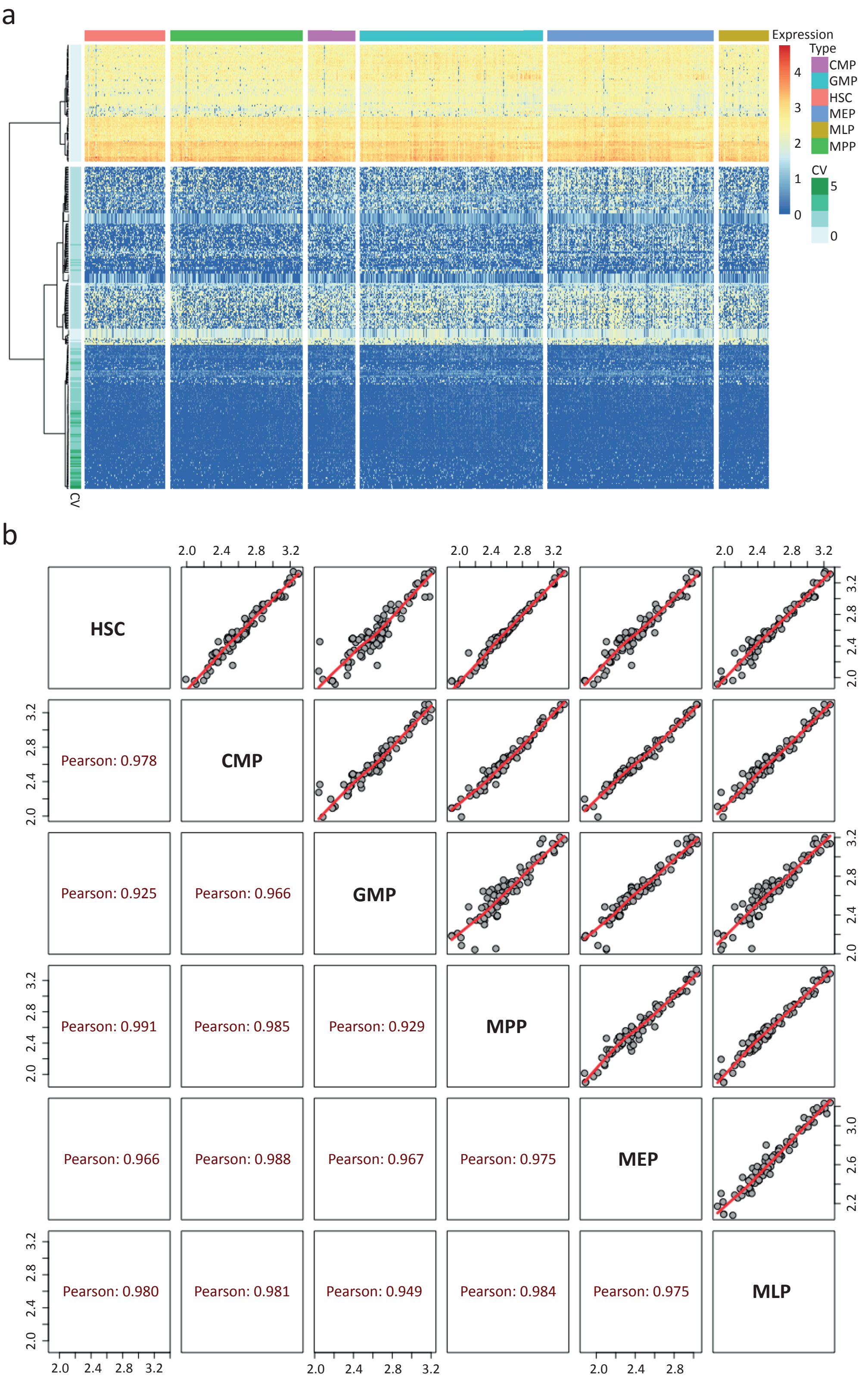

Supplementary Figure 9. Analysis of ribosomal genes in human HSCs and progenitors. a) Heatmap of ribosomal gene expression in human HSCs and progenitors. Clustering of all human ribosomal genes across different HSPC populations, using Euclidean distance and Ward Linkage. On the top, cytosolic ribosomal genes are shown, whereas the bottom cluster consists of mitochondrial ribosomal genes. On the left hand side, the calculated coefficient of variation (CV) across cells is presented for each gene. b) Correlation analysis of expressed ribosomal genes across 891 HSPCs. Pairwise Pearson correlation revealed similar expression levels of the expressed cytosolic ribosomal genes in HSC and various progenitors (MPP, CMP, GMP, MEP and MLP).

Supplementary Table 1: Detailed description of fish lines. Details of transgenic and wild type lines used for single cell experiments, giving age, number of fish, number of 96 well plates sorted, number of cells passing quality control and references.

Transgenic line	Age (months)	No fish	No Plates	No cells	Reference
Tg(cd4:mCherry)	5	1	1	23	Dee *et al*. 2016
Tg(cd41:EGFP)	6-13	4	6	519	Lin *et al.* 2005
Tg(gata1a:GFP)	6	1	1	63	Long *et al.* 1997
Tg(lyz:DsRed2)	11	1	1	31	Hall *et al.* 2007
Tg(mfap4:tdTomato)	7	1	2	140	Walton *et al.* 2015
Tg(mpx:EGFP)	10	1	2	133	Renshaw *et al.* 2006
Tg(runx1:mCherry)	7	1	2	160	Tamplin *et al.* 2015
Tg(tal1:EGFP)	11	1	1	74	Zhang & Rodaway 2007
WT_P1*	6	1	1	57	NA
WT_P2*	6	1	1	31	NA
WT_P3*	6	1	1	22	NA
WT_P4*	6	1	1	84	NA
WT_P5*	1	1	1	85	NA

*Tubingen Long Fin

Supplementary Table 2. FACS markers at different human cell types. Detailed description of the Human progenitor populations considered in the present study with their respective FACS markers.

Cell Type	Number of Cells	FACS markers
HSC	109	CD34+ CD38- CD45RA- CD90+ CD49f+
MPP	179	CD34+ CD38- CD45RA- CD90-
CMP	64	CD34+ CD38+ CD10- CD45RA- CD135+
GMP	247	CD34+ CD38+ CD10- CD45RA+ CD135+
MEP	224	CD34+ CD38+ CD10- CD45RA- CD135-
MLP	68	CD34+ CD38- CD45RA+
