# Supplementary material for: Single-cell RNA-sequencing uncovers transcriptional states and fate decisions in haematopoiesis
Source: Nat Commun. 2017 Dec 11;8:2045. doi: 10.1038/s41467-017-02305-6 (PMC5725498; doi:10.1038/s41467-017-02305-6)
Supplement: Supplementary file 3 — Description of Additional Supplementary Files [file 41467_2017_2305_MOESM3_ESM.pdf]

Description of Additional Supplementary Files

File Name: Supplementary Data 1
Description: Lists of genes using different processing strategies. Full list of the DE expressed genes in each of the five Monocle states; GO term enrichment analysis for each of the states; dynamically expressed genes in monocytes, neutrophils, erythrocytes and thrombocytes; expression of ribosomal genes which show dynamic and random expression in pseudotime in monocytes, neutrophils, erythrocytes and thrombocytes.
